# Silymarin: a promising modulator of apoptosis and survival signaling in cancer

**DOI:** 10.1007/s12672-025-01800-3

**Published:** 2025-01-21

**Authors:** Ujjawal Sharma, Praveen Kumar Sahni, Bunty Sharma, Madhu Gupta, Damandeep Kaur, Darin Mansor Mathkor, Shafiul Haque, Sabiha Khatoon, Hardeep Singh Tuli, Astha Mishra, Faraz Ahmad

**Affiliations:** 1 Department of Human Genetics and Molecular Medicine, Central University of Punjab, Bhatinda, 151001 India; 2 Department of Biotechnology, Graphic Era (Deemed to Be University), Dehradun, Uttarakhand India; 3 Department of Pharmaceutics, School of Pharmaceutical Sciences, Delhi Pharmaceutical Sciences and Research University, New Delhi, 110017 India; 4 University Center for Research & Development (UCRD), Chandigarh University, Gharuan, Mohali, Punjab 140413 India; 5 Department of Nursing, College of Nursing and Health Sciences, Jazan University, 45142 Jazan, Saudi Arabia; 6 Universidad Espiritu Santo, Samborondon, Ecuador; 7 University of Oklahoma Health Sciences Center, Oklahoma City, OK 73104 USA; 8 Department of Bio-Sciences and Technology, Maharishi Markandeshwar Engineering College, Maharishi Markandeshwar (Deemed to Be University), Mullana, Ambala, 133207 India; 9 Department of Optometry, Chitkara School of Health Sciences, Chitkara University, Rajpura, Punjab India; 10 Department of Biotechnology, School of Bio Sciences and Technology (SBST), Vellore Institute of Technology (VIT), Vellore, 632014 India

**Keywords:** Anticancer, Apoptosis, Natural compounds, Silymarin, Therapeutics

## Abstract

Cancer, one of the deadliest diseases, has remained the epicenter of biological research for more than seven decades. Yet all the efforts for a perfect therapeutic cure come with certain limitations. The use of medicinal plants and their phytochemicals as therapeutics has received much attention in recent years. Silymarin, a polyphenolic flavonoid with a variety of anti-cancerous properties, was isolated from the plant *Silybum marianum*. The present review centres on the function of silymarin in controlling important signalling pathways related to apoptosis and survival, such as the JAK/STAT pathway, PI3K/Akt/mTOR, Bcl-2/Bax, and Fas/FasL. It is emphasised that silymarin's capacity to target these pathways is a key mechanism behind its anticancer effects against a variety of malignancies. By upregulating pro-apoptotic and downregulating anti-apoptotic proteins, silymarin controls a series of events that result in tumor suppression and cell death in a variety of cancer types. The low bioavailability and limited therapeutic efficacy of silymarin are improved by the application of various nano-delivery systems. As efficient carriers, liposomes, polymeric micelles, lipid- and metal-based nanoparticles, increase the solubility and distribution of silymarin in target tissues. Lastly, a number of preclinical studies that provide a basis for upcoming therapeutic interventions are highlighted in the review, providing encouraging directions for additional research and advancement.

## Introduction

Cancer remains a significant global health concern, with a staggering ten million human lives lost to the disease in 2022 [1]. This sobering statistic highlights the urgent need to address this complex and multifaceted condition. While recent advancements in stem cell therapy, nanoparticle technology, and targeted treatment approaches have offered promising alternatives, traditional methods involving radiation, chemotherapy, and surgical intervention continue to play a crucial role in the fight against cancer [2]. These procedures, while helpful, have a number of negative effects and expensive treatment costs. An alternate, less complex treatment approach is urgently needed. Traditional treatment approaches have drawn a lot of attention lately. Among these approaches is phytotherapy, also known as phytomedicine, which involves treating illnesses with plants and plant extracts. The body's capacity to heal and defend itself may be restored by phytomedicine [3]. Amidst the diversity of traditionally used plants, *Silybum marianum*, also known as ‘Milk thistles’, is one of the most exploited plants for its various medicinal properties and treatment of several diseases, including cancer [4]. The foremost bioactive compound of the plant is Silymarin, which is acquired from the seeds of the plant and has been used as a natural medicine for over 2000 years to protect the liver in opposition to diseases like hepatitis and cirrhosis, because of its anti-inflammatory, anti-oxidative, and immunomodulatory homes [5]. Moreover, in vivo and in vitro studies have highlighted the anti-proliferative and chemo-preventive capability of silymarin in different types of cancer [6]. Silymarin can result in apoptosis, inhibit tumor cellular growth and angiogenesis, and modify the cellular cycle and immune response towards cancer. Additionally, studies performed in vitro and in vivo have validated the anti-proliferative and chemopreventive capacity of silymarin in numerous cancer types [6]. Silymarin can trigger apoptosis, impede the growth and angiogenesis of tumor cells, and control the cell cycle and immune response against cancer [7]. The tumor-inhibiting properties of silymarin stem from its ability to modulate many cell signaling pathways such as Bcl-2/Bax, PI3K/Akt/mTOR, STAT3, and MAPK pathway [7]. Furthermore, silymarin’s poor bioavailability and limited thérapeutic efficacy have been overcome by encapsulation of silymarin into nanoparticles [5].

Although silymarin's therapeutic potential has been thoroughly investigated in the past, the novelty of the review lies that it offers a thorough examination of the chemistry and pharmacokinetics of silymarin while highlighting its unique function in regulating several signaling pathways linked to apoptosis and cell survival in different types of cancer. The review combines the results of several recent research, emphasizing the interrelated process by which silymarin provides its therapeutic effects, providing a better understanding of impact of silymarin on key pathways like Bcl-2/Bax, PI3K/Akt/mTOR, STAT3, and MAPK. Opportunities related to the ability of those trends in nano-delivery systems to improve the compound's bioavailability and effectiveness in cancer therapy can also be highlighted.

## Chemistry and pharmacokinetics

**Fig. 1:**
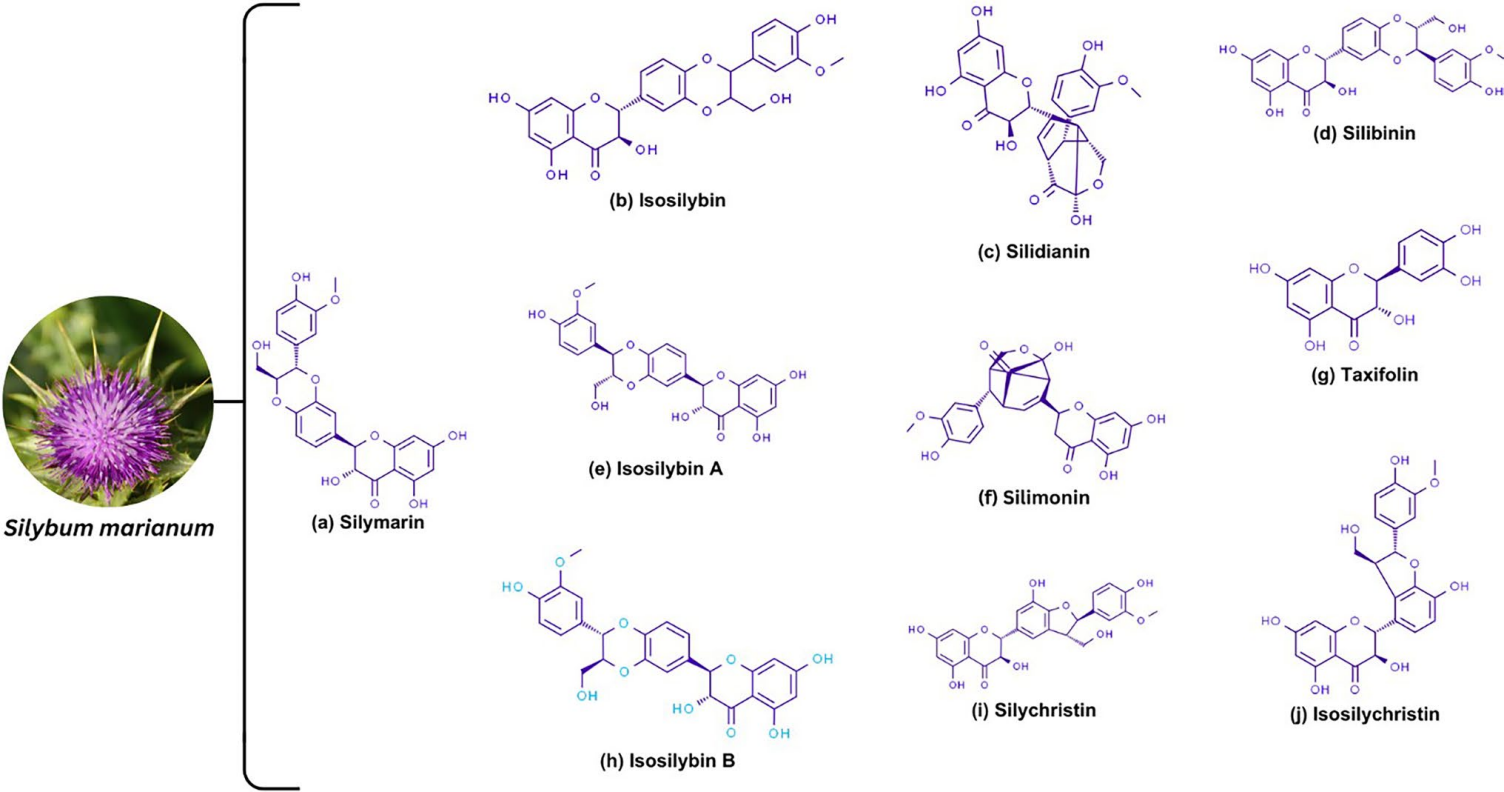
Chemical structure of silymarin and its constituents. The figure shows the principal bioactive compound of *Silybum marianum* (**a**) Silymarin and its other constituent flavonolignans and flavonoids (**b**–**j**)

Silymarin is a phytochemical compound extracted from the plant *Silybum marianum*, commonly known as milk thistle. This polyphenolic flavonoid extract consists of a complex mixture of flavonolignans, fatty acids, and other polyphenolic components (Fig. 1) [8]. Among the flavonolignans, silybin (or silibinin) is the most active and abundant compound, accounting for 60–70% of the total silymarin content. Silibinin exists in two diastereomeric forms, silibinin A and silibinin B [4]. The other flavonolignans include silychristin (20%), silydianin (10%), and isosilibinin (5%). Isosilibinin also exists as two diastereomers, isosilibinin A and isosilibinin B. Other flavonolignans include isosilychristin and silimonin. Apart from the significant flavonolignans, an essential flavonoid, taxifolin is also present in silymarin [4].

Silymarin is commonly consumed orally. After ingestion, gastrointestinal tract (GIT) cells rapidly absorb it in all regions of the small intestine. The highest absorption occurs in the duodenum, followed by the jejunum, ileum, and colon [9]. Upon entering the small intestine, silymarin is rapidly conjugated primarily by glucuronidation. The conjugation process continues in the liver. Despite its rapid uptake by GIT cells, the bioavailability of silymarin is very low [10]. Silymarin is barely 20–50% absorbed by the GIT cells and has an absolute oral bioavailability of 0.95% [11]. Multiple factors contribute to its low bioavailability, including poor drug solubility, instability in gastrointestinal fluids, difficulty crossing intestinal barriers, formulation dissolution difficulties, early metabolism in the intestine and liver, and efflux transporter activity [12]. However, two well-defined reasons account for this notably low oral bioavailability. First, the intestinal cells efflux out the free and conjugated forms of silymarin. Second, they are secreted into bile by the liver. The flavonolignan silibinin undergoes phase I and phase II metabolism with multiple conjugations in individuals in phase II. Phase I metabolism is minimal, while the conjugation achieved by phase II metabolism is a highly relevant metabolic step. It is mainly excreted by the liver and bile due to an active transport mechanism [12]. The highest proportion of flavonolignans is excreted as feces, which is primarily due to biliary excretion and the movement of unabsorbed and effluxed compounds through GIT [12]. The low bioavailability of silymarin can be enhanced by the use of various delivery systems like nanostructured systems, self-micro emulsifying drug delivery systems (SMEDDS), phytosome complexes, and natural bioenhancers. These methods increase solubility and bioavailability, eradicating the drawbacks of silymarin and increasing its availability in clinical use. The nano delivery systems have been discussed in detail in the following sections.

## Cell death and survival signalling modulation in cancer

Apoptosis is a programmed cell death mechanism that helps to eliminate damaged or malignant cells. Phytochemicals can affect cancer cell survival by affecting apoptosis-related pathways. They can either induce apoptosis in cancer cells by activating pro-apoptotic proteins (such as Bax and caspases) or inhibit survival pathways (such as PI3K/Akt), resulting in cancer cell death and reduced tumor development. These phytochemicals frequently regulate molecular pathways associated with cancer growth and progression. The specific processes include enhancing antioxidant status, inactivating carcinogens, suppressing proliferation, inducing cell cycle arrest and apoptosis, and regulation of immune system [13].

### Fas/FasL pathway

**Fig. 2:**
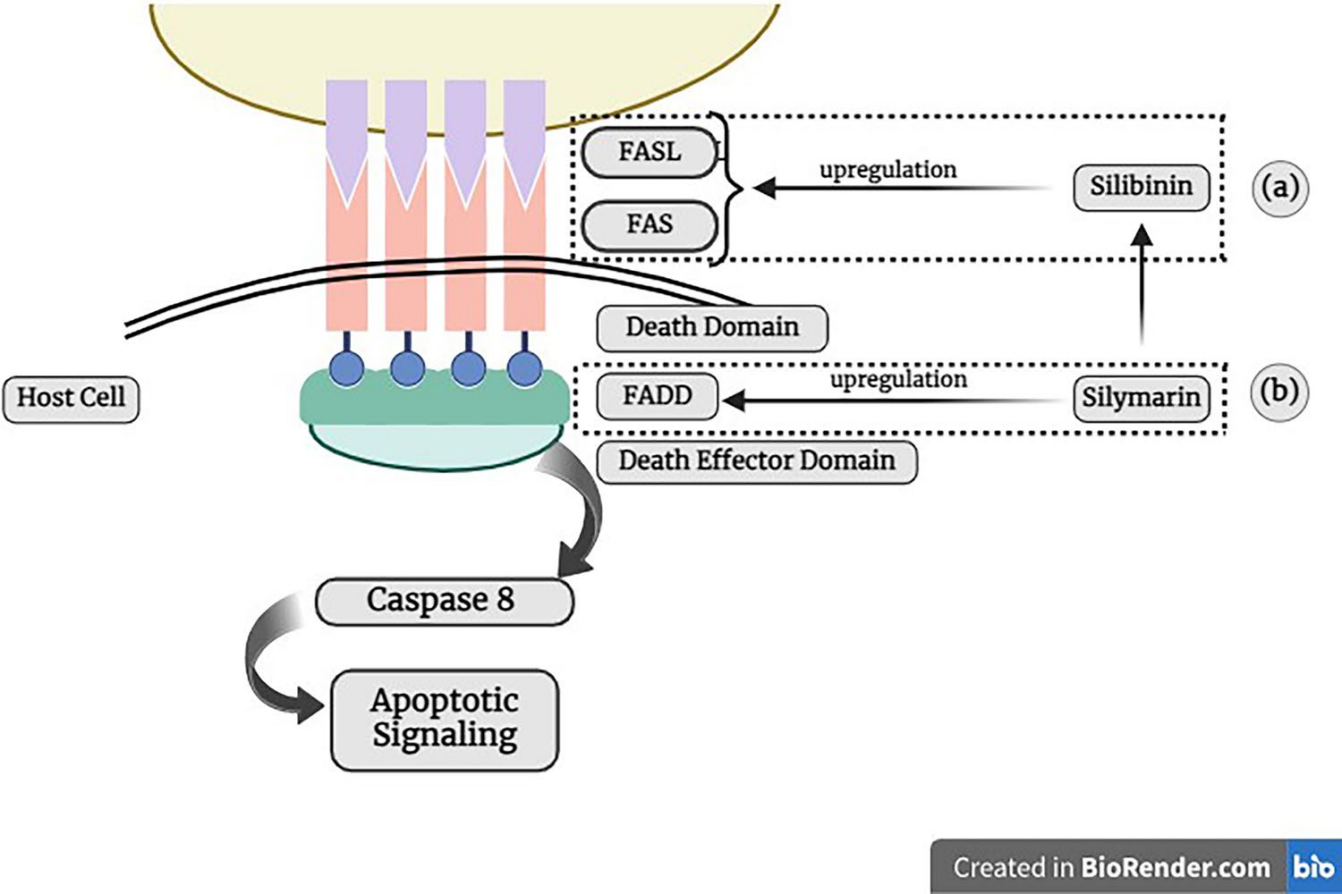
Modulation of the Fas/FasL Pathway by Silymarin. **a** Silibinin enhances the Fas pathway in cancer cells by upregulating the expression of Fas and FasL. This activation leads to initiating downstream apoptotic signaling. **b** Silymarin induces apoptosis by upregulating the expression of FADD, a downstream component of the death receptor pathway. This upregulation leads to the cleavage of procaspase 8 and the initiation of apoptotic cell death

In several research, silymarin has proven the capability to regulate the Fas/FasL pathway, a key apoptosis regulator. In human melanoma cell line A375-S2 treated with CH11, silymarin triggered apoptosis via upregulating the expression of FADD (Fig. 2b), a downstream component of the death receptor pathway, subsequently leading to the cleavage of procaspase 8 and initiation of apoptotic cell death [14]. Silymarin, especially its principal active constituent silibinin, enhances the Fas pathway in most cancers cells by upregulating the Fas and Fas L (Fig. 2A). This activation forms the Fas-FADD-caspase 8 complex, initiating downstream apoptotic signaling. By upregulating Fas and Fas L expression, silymarin promotes apoptosis through the death receptor-mediated pathway, contributing to its anticancer effects [15]. Another study demonstrated the effect of silibinin on MCF-7 cells (human breast cancer cell line). Silibinin upregulated the expression of FasL and FADD in MCF-7 cells [16]. In MCF-7 cells that were untreated with silibinin, caspase existed in two isoforms, 55 kDa and 53 kDa. The treatment with silibinin cleaved the pro-caspase 8 into two active forms of 43 kDa and 41 kDa.silibinin. The cleavage of pro-caspase-8 suggests activation of the extrinsic apoptotic pathway via the Fas/FasL signaling cascade. Furthermore, in cells treated with silibinin, the insulin-like growth factor 1 receptor inhibitor AG1024 raises the expression of FADD. This increased expression of FADD leads to the reinforcement of silibinin-induced apoptosis, highlighting the complex relation between the Fas/FasL pathway and IGF-1R inhibition in regulating apoptotic pathways caused by silibinin treatment. Silibininsilibinin [16].

### Bcl-2/Bax pathway

Silymarin exerts its anticancer effects by modulating Bcl-2 family proteins, which play critical role in the intrinsic cell death pathway. Bax (pro-apoptotic) and Bcl-2 (anti-apoptotic) are two important proteins in the Bcl-2 family that support and inhibit the cell death pathway by regulating mitochondrial function, mitochondrial membrane permeability and cytochrome release [17]. The ratio of Bax to Bcl-2 acts as an apoptotic switch which allows the cell to determine fate. Increasing the Bax/Bcl-2 ratio decreases the cell's resistance to apoptotic signals and causes increased cell death and fewer tumors [18]. In a study involving breast cancer cells, MCF-7 and MDA MB 231, the cell proliferation and viability assay was carried out, with results indicating an increase in levels of Bax by silymarin. In contrast, the expression of Bcl-2, an anti-apoptotic protein, was decreased [19]. A similar former study on the treatment of silymarin and silibinin on p53 + / + fibroblasts and JB6 C141 cells (preneoplastic epidermal keratinocytes) revealed a notable change in the Bax/Bcl-2 ratio favouring apoptotic signaling in cells treated with silymarin. This shift led to the cleavage of caspase-3 and poly (ADP-ribose) polymerase and an increase in apoptotic protease-activating factor 1 and cytochrome c levels [20]. Bax is also upregulated and leads to the activation of caspase-3. These effects appear to be associated with the PI3K pathway. Silibinin inhibits the PI3K activity, leading to the reduction of FoxM1 (Forkhead box M1) and the subsequent activation of the mitochondrial apoptotic pathway [21]. Silymarin’s ability to target Bcl-2 family proteins suggests that it may be a promising therapeutic agent for cancer treatment by increasing apoptosis and inhibiting cancer cell survival pathways.

### PI3K/Akt/mTOR pathway

The PI3K/Akt/mTOR pathway regulates several significant cellular processes, including cell cycle, cell proliferation, and apoptosis. The PI3K/Akt/mTOR pathway stands out as a frequently activated pathway within cellular signaling networks and mediates the balance between normal and neoplastic cells [22]. In glioblastoma multiforme, taxifolin, a flavonoid in silymarin, has been observed to inhibit the mTOR/PI3K and promote autophagy. In an in-silico study, taxifolin could bind to the catalytic of PI3K and rapamycin site of mTOR. In another in vitro experiment on five glioma cells, taxifolin suppressed the activity of PI3K and mTOR [23]. An in vivo study on artificially induced hepatocellular carcinoma in rats revealed that silymarin could inhibit the growth of cancer cells by suppressing the PI3K/Akt/mTOR pathway [24]. The effects of silibinin on the mTOR pathway in cervical cancer cells have also been explored. The afore mentioned studies indicated that silibinin interferes with mTOR signaling by inhibiting phosphorylation of several key components in this pathway, such as mTOR, p70S6K and 4E-BP1 [25]. The mTOR pathway signaling in turn may result in low levels of HIF-1α due to the unfavorable conditions of hypoxia. It is also worth noting that apart from the inhibition of mTOR signaling, silibinin activates the Akt pathway in cervical cancer cells. This activation of Akt could have some bearing on the overall antitumor activity of silibinin in cervical cancer cells. According to the data available, one of the consequences that resulted in permanent silibinin-induced inactivation of typically proliferative signaling in cervical cancer cells requires the inhibition of mTOR pathways [25], directed at the mTOR pathways and adjusting the activity of Akt, silibinin proves to be a promising drug in the direction of suppressing the tumor and increasing the number of cell deaths. These findings provide insight into molecular approach explaining the anticancer potential of silibin.

### JAK/STAT pathway

**Fig. 3:**
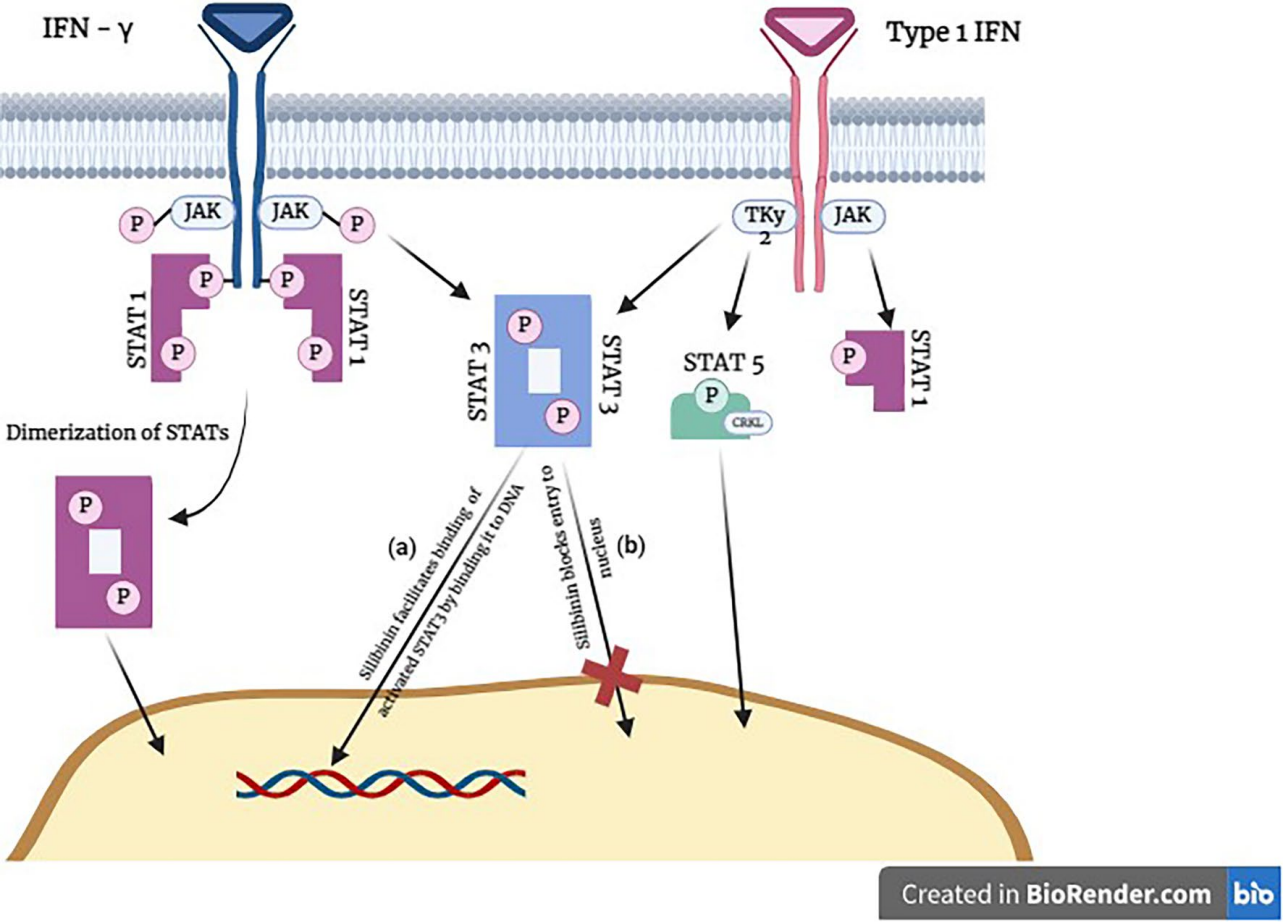
Inhibition of STAT3 Signaling by Silibinin. Silibinin inhibits STAT3 signaling through multiple mechanisms. **a** Silibinin blocks activated STAT3 by binding it to DNA, suppressing STAT3-directed gene expression. **b** Furthermore, Silibinin prevents activated STAT3 from entering the nucleus

JAK/STAT is another important intracellular signaling pathway responsible for many cellular physiological processes. including cell growth and proliferation [26]. JAK/STAT activation may promote tumor cell proliferation [27]. In a study on diabetic nephropathy, silymarin was able to regulate the JAK/STAT signaling pathway by reducing the expression of pSTAT3 and pJAK2, thereby potentially altering cell death signals [26]. Silibinin also inhibits STAT3 by preventing STAT3 from entering the nucleus (Fig. 3b). Silibinin blocks activated STAT3 by binding it to DNA and suppressing STAT3-directed gene expression (Fig. 3a) [28]. In another study on lung cancer, silibinin inhibited STAT3, HIF-1α, and NF-κB, thereby reducing the population of lung macrophages and limiting angiogenesis [29]. A recent discovery reveals that silibinin delays the progression of endometrial carcinoma via inhibiting STAT3 activation and lowering lipid accumulation, which is regulated by SREBP1 [30]. Sorafenib and silibinin work together to target both liver cancer cells and cancer stem cells. This combination operates by suppressing the STAT3/ERK/AKT pathways and decreasing the production of Mcl-1 and Bcl-2 proteins [31]. Silibinin therapy reduced fibronectin expression, which is stimulated by epidermal growth factor, in triple-negative breast cancer cells. This decrease in fibronectin expression led to the deactivation of STAT3 [32]. Silibinin induced cell cycle arrest and apoptosis in gastric cancer cells by activating the STAT3 pathway. This effect was evidenced by reducing the expression of CDK1, survivin, Bcl-xL, cyclinB1 and Mcl- 1 and simultaneously activate caspases 3 and 9 [33]. In a separate investigation, silibinin and its 2,3-dihydro derivative decreased phosphorylation of STAT3 and Akt in cancer cells. Additionally, it hindered the activation of transcription factors NF-κB and AP-1 these data suggest that silymarin and its components may have potential as anticancer agents [34].

## Nano delivery aspects of silymarin

Silymarin possesses biological activity that could improve human health and well-being. However, its potential as a better therapeutic agent is limited by its poor bioavailability and low water solubility. Nano-delivery systems hold the potential to overcome these restrictions by improving silymarin’s functional performance [35]. For this reason, there has been great interest in using nanocarriers to improve their water solubility, chemical stability and absorption of silymarin. This strategy aims to expand the use of silymarin in functional foods, nutritional supplements and medicines. Silymarin can be delivered to cells by nanocarrier systems such as liposomes, polymeric micelles,lipid nanoparticles (NPs) and metallic NPs [5]. Liposomes, bilayer vesicles with amphiphilic characteristic areable to transport hydrophilic and hydrophobic drugs [36]. Polymeric micelles are known for their biocompatibility and cancer targeting ability. It is ideal for the encapsulation of lipophilic molecules [37]. Studies have shown that silymarin-loaded liposomes and polymeric micelles improve bioavailability and therapeutic efficacy in cancer treatment [38, 39]. In addition, lipid-based nanoparticles have increased the oral bioavailability of silymarin [40]. Phospholipid-based phytosomes were prepared to increase the oral bioavailability of silymarin. The improved silymarin phytosome formulation significantly increases the water solubility of silymarin and the drug release in vitro for up to 24 h, surpassing the conventional drugs. Moreover, it improved silymarin's oral bioavailability, exhibiting a sixfold rise in systemic absorption compared to pure silymarin [41].

Among the nanoparticles that have been explored for drug delivery, the biodegradable nanoparticles are of special significance [42]. Depending on the base materials, such as poly(lactic-co-glycolic acid) (PLGA), Poly(HydroxyButyrate-co-HydroxyValerate) (PHBHV), chitosan these NPs are biocompatible and further dissipate in the body into non-toxic products [43]. For example, PLGA-based nanoparticles demonstrated better features in encapsulating silymarin using nanoparticles where the release and protection of the drug is well controlled and shielded from degradation [44]. PHBHV–based nanocarriers (~ 100 nm) have demonstrated high potential in colon cancer by enhancing cellular internalization and improved tumor penetration which is indicated by the small size of multicellular tumor spheroids in models [45]. In addition, silymarin encapsulated within PHBHV nanocarriers presented profound cytotoxicity against HT-29 colorectal carcinoma cells where cell viability was reduced over 6 to 24 h of treatment [45]. Similarly, silymarin loaded chitosan nanoparticles have been studied for oral application due to mucoadhesive action of chitosan that provided better solubility and bioavailability. Liang et al., created a chitosan-based lipid polymer hybrid nanoparticles that boosted the bioavailability of silymarin by 14.38-fold and cured liver functions and lowered triglyceride levels effectively making the hybrid nanoparticles effective nanocarriers [46].

Additionally, there is potential for improving therapeutic efficacy and bioavailability with metal-based nanoparticles (NPs). For instance, studies assessing the tumor-killing potential of silibinin-loaded synthetic nanoparticles, PLGA-PEG-Fe3O4, on the breast cancer cell line T47D. These silibinin-loaded nanoparticles hindered the growth of T47D cells in a dose-dependent manner and dramatically reduced telomerase gene expression [41]. In a different study, Jackson et al. discovered that silibinin-loaded silver nanoparticles could induce apoptosis-related morphological changes in a colorectal cancer cell line via a p53-dependent route [47]. Many studies confirm the improved efficacy of silymarin-loaded NPs for their anti-cancer effects, as described below in Table 1. Furthermore, mixing silymarin with other natural products or medicinal drugs also improves its anticancer activity [48].

**Table 1:** Anti-tumor effects of Silymarin-loaded nanoparticles in in-vitro and in-vivo studies

Product Name	Cancer type	Cell line/model	Findings	References
Silymarin-loaded zein nanoparticles (SLNPs)	Breast cancer	MCF-7	1. Inhibited survival and growth of MCF-7 cells 2. Enhanced cytotoxic effects on MCF-7 cells 3. Induced cell cycle arrest at G2/M checkpoint	[49]
Silymarin nanoliposomes	Breast cancer	In-vitro 4T1 mouse breast cancer cell In-vivo Female BALB/c mice	1. Combination of silymarin nanoliposomes with iron exhibited cytotoxic effects on 4T1 mouse breast cancer cells 2. The mechanism of action may involve the prooxidant activity of silymarin in combination with iron, which could lead to increased oxidative stress in cancer cells, ultimately contributing to their cytotoxicity 3. Combination was effective in inducing tumor shrinkage, delaying tumor growth, and promoting tumor tissue necrosis	[50]
Genipin-crosslinked albumin nanoparticles containing neratinib and silibinin	Breast cancer	MDA-MB-231 and 4T1	1. Nanoparticles showed enhanced uptake and cytotoxicity against triple-negative breast cancer (TNBC) cells 2. These nanocarriers induced a mixed type of cell death, combining apoptosis and ferroptosis pathways	[51]
Silymarin-Loaded Tin(IV) (SM-Sn)Nanoparticles	Colorectal cancer	SW480	1. SM-Sn nanoparticles increased intracellular ROS level and malondialdehyde content 2. The NPs also induced cell cycle arrest and apoptotic gene expression	[52]
Silymarin and Metformin Dual-Loaded in Mesoporous Silica Nanoparticles (MSNs)	Breast cancer	MCF7MX and MCF7	1. Co-loaded silymarin and metformin in MSNs enhanced chemotherapy efficacy against breast cancer cells 2. The nanoparticles increased sensitivity to mitoxantrone and induced apoptosis, indicating synergistic effects of the drugs delivered via MSNs	[53]
Silymarin loaded in nanostructured lipid carriers and incorporated in mucoadhesive in-situ gel (SME-NLCs-Plx/CP-ISG)	Oral cancer	KB	1. SME-NLCs-Plx/CP-ISG induced apoptosis at Sub-G0 phase	[54]
Silymarin-mediated selenium nanoparticles (SeNPs)	–	RAW264.7 cells	1. Si-SeNPs exhibit higher anti-inflammatory activities toward RAW264.7 cells compared to silymarin 2. SeNPs suppressed LPS-induced NF-κB activation by potentially downregulating the PI3K/Akt signaling pathway	[55]
Silibinin-Loaded Nanostructured Lipid Carriers (NLC)	Ovarian cancer	A2780	1. Silibinin-loaded NLC-sensitized cisplatin-resistant ovarian cancer cells and showed a synergistic inhibitory effect	[56]
SB-loaded PCL/Pluronic F68 nanoparticles	Lung cancer	In-vitro A549 In-vivo Sprague–Dawley rats	1. The Nanoparticles induced apoptosis, cell cycle arrest, and significant inhibition of tumor growth in lung cancer models	[57]
Selenium-mediated silymarin nanoparticles (Si-SeNPs)	Gastric cancer	AGS cells	1. Si-SeNPs demonstrated increased cytotoxicity against AGS gastric cancer cells and induced apoptosis and autophagy pathways while inhibiting PI3K/AKT/mTOR signaling	[25]

## Safety studies

Silymarin, though obtained from plant sources, can be considered safe; extensive research is required till its clinical application. To establish its safety, Silymarin has been tested at different concentrations in clinical as well as in animal experiments. For example, in an in vivo experiment, doses of 2, 4 and 20 mg/kg of body weight of silymarin, were administered to mice, and there was no chromosomal abnormality found in the cells of bone marrow, and therefore, it proved to be safe even at higher doses [58]. Silymarin creams and gels have been well-tolerated in individuals with illnesses such as melasma and gastrointestinal cancer, with no known side effects at concentrations routinely employed in these formulations [59, 60]. In other in vivo tests to assess the safety and toxicity of silymarin, male and female mice and rats were given differing amounts of silymarin over different time periods. This research sought to determine the effects of silymarin on body weight, blood parameters, and probable toxicity. The results showed that silymarin administration had no significant effect on the animals' body weight or blood parameters, confirming its safety profile [61]. While silymarin has demonstrated good safety profiles in both animal and human studies, further study is needed to completely understand its long-term effects, potential drug interactions, and any specific toxicities that may occur in certain situations or groups. Table 2 highlights various in vivo and in vitro studies of silymarin on different malignancies and their possible anti-cancerous effects.

**Table 2:** various in vivo and in vitro studies of silymarin on different malignancies and their possible anti-cancerous effects

Cancer	Study type	Cell line/model	Effects	Mechanism	Concentration	References
Ovarian cancer	In-vitro	A2780 cells	Anti-proliferative, increased apoptosis	↓ miR-27a, ↓ miR-155	0–500 µM/mL	[62]
Hepatocellular carcinoma	In-vitro	HepG2 and Hep3B cells	Anti proliferative, induced apoptosis	↓ HIF-1α/VEGF signaling	58.46 and 75.13 μmol/L	[63]
Fibrosarcoma	In-vitro	HT1080 cells	Inhibits cell invasion	↓ MMP-2, ↓ IL-1β, ↓ ERK-1/2, ↓ p-p38	25 μM	[64]
Human acute promyelocytic	In-vitro	NB4 cells	Enhances apoptosis	↓ Survivin, ↓ Bcl-2, ↑ Bax	–	[65]
Breast cancer	In-vitro	T47D and MCF-7 cells	Induce apoptosis and cell cycle arrest	↓ MiR‑20b, ↑ BCL2L11, ↑ PTEN, ↑ Caspase 9	50, 75,100, 150, 200, and 250 μM	[66]
Human epithelial colorectal adenocarcinoma	In-vitro	CaCo-2	Antiproliferative and anti-inflammatory	↓ IL-1, ↓IL-6, ↑ TGF-β	5, 10, 20, 40, and 80 µM	[67]
Ehrlich solid tumor	In-vivo	Female Swiss albino mice	Anti-angiogenic, enhance apoptosis, inhibit cell division	↓ VEGF, ↓ NF-κβ, ↑ E-cadherin	200 mg/kg body weight	[68]
Basal cell carcinoma	In-vivo	Ptch^tm1Mps^/J mice	Inhibits initiation and BCCs and other skin lesions induced by UVB exposure	↓ Smo, ↓ Gli1, ↑ Ck14, ↑ Ck15	9 mg	[69]
Hepatocellular carcinoma	In-vivo & In-vitro	Wistar rats HepG2 and Huh7 cell lines	Anti-hepatocarcinogenic	↓ HGF, ↓ c-Met, ↓ Wnt, ↓ β-catenin, ↓ PI3K, ↓ Akt, ↓ mTOR	200 mg/kg body weight (bw), 150 mg/kg bw, 5 mg/kg bw 250, 125, 62.5, 31.25, 15.6, 7.8, 3.9, 2, and 1 μg/mL	[24]
Lung cancer	In-vitro	A549, H460, H292	Induce cell cycle arrest and apoptosis, inhibit angiogenesis, cell migration, and invasion	↓ EGFR, ↓ STAT5 ↓ AKT	50, 100, 200, 300, and 400 µM	[70]

## Conclusion

Natural bioactive compounds are becoming increasingly famous as a method of treating diseases like cancer. A promising therapeutic alternative for the treatment of cancers can be silymarin, derived from milk thistle. What makes it appealing is its potential to target significant apoptotic and survival signaling pathways, including Fas/FasL, Bcl-2/Bax, PI3K/Akt/mTOR, and even JAK/STAT. Silymarin's ability to modulate these pathways promotes not only apoptosis, but also inhibits tumor growth, angiogenesis, and metastasis in a number of cancer types. Silymarin has also shown promising antitumor effects in the treatment of tumor growth in various animal and in vitro models.

The development of nano delivery systems has resulted in substantial advances in silymarin research, with demonstrated ability to improve bioavailability, solubility, and therapeutic efficacy. Nano formulations such as liposomes, polymeric micelles, and lipid and metal-based nanoparticles have demonstrated exceptional silymarin cellular absorption and targeted delivery. Overall, these systems have the potential to dramatically increase silymarin's anti-cancer effects while preserving a longer release time and greater stability. These findings were validated in preclinical experiments, which demonstrated that nano encapsulated silymarin formulations induce apoptosis, regulate immune response, and suppress cancer growth.

Silymarin as a natural therapeutic agent has been demonstrated to be safe and effective through numerous in vitro and in vivo studies. Thus, silymarin's widespread use in traditional medicine, along with recent pharmacological advances, make it a promising candidate for cancer treatment.

## Limitation and future perspectives

Despite the pharmacological potential of silymarin, its limited solubility and low bioavailability has weighted down its therapeutic potential. Liposomes and other nanoparticle-based delivery techniques has proven to overcome these drawbacks. Yet, there are a couple of challenges in presenting nanoformulated or readily available forms of silymarin as an encouraging prospect for cancer therapy. However, modifying formulation factors such as particle size, surface charge, and drug loading capacity may improve the performance of these nanocarriers.

Moreover, there are limited clinical trials to validate experimental findings, primarily large-scale human investigations. Both preclinical and in vitro studies suggest very promising outcomes, but these need to be confirmed by more robust clinical trials, also assessing the safety and efficacy of silymarin based treatments in humans. A significant concern is a lack of understanding of the potential risks linked with silymarin in people. More research is needed to determine the anti-tumor effects of silymarin and its active components in therapeutic applications. Additionally, preclinical and clinical studies are required to assess silymarin-loaded nanoparticles' safety, effectiveness, and pharmacokinetics in cancer patients.

## Data Availability

No datasets were generated or analysed during the current study.
